# Release of Antibiotics Out of a Moldable Collagen-β-Tricalciumphosphate-Composite Compared to Two Calcium Phosphate Granules

**DOI:** 10.3390/ma12244056

**Published:** 2019-12-05

**Authors:** Klaus Edgar Roth, Gerrit Steffen Maier, Irene Schmidtmann, Ulrich Eigner, Wolf Dietrich Hübner, Fabian Peters, Philipp Drees, Uwe Maus

**Affiliations:** 1 Zentrum für Orthopädie und Unfallchirurgie, Unimedizin, 55131 Mainz, Germany; Philipp.Drees@unimedizin-mainz.de; 2 Pius Hospital, Universitätsklinik für Orthopädie und Unfallchirurgie, 26121 Oldenburg, Germany; gerrit.s.maier@gmx.de (G.S.M.); uwemaus@online.de (U.M.); 3 Institut für Medizinische Biometrie, Epidemiologie und Informatik, Unimedizin Mainz, 55131 Mainz, Germany; ischmidt@uni-mainz.de; 4 Labor Limbach, 69126 Heidelberg, Germany; ulrich.eigner@labor-limbach.de; 5 Curasan AG, 65933 Frankfurt am Main, Germany; wolf-dietrich.huebner@curasan.de (W.D.H.); fabian.peters@curasan.de (F.P.); 6 Klinik für Orthopädie und Unfallchirurgie, Universitätsklinikum Düsseldorf, 40225 Düsseldorf, Germany

**Keywords:** bone substitute, antibiotics, elution rate, foam, controlled drug release, Minimum Inhibitory Concentration (MIC), Minimum Biofilm Eradication Concentration (MBEC)

## Abstract

Bacterial bone infections after revision surgeries and diseases, like osteomyelitis, are still a challenge with regard to surgical treatments. Local bone infections were treated with antibiotics directly or by controlled drug-releasing scaffolds, like polymethylmethacrylate (PMMA) spheres, which have to be removed at a later stage, but there is a risk of a bacterial contamination during the removement. Therefore, biomaterials loaded with antibiotics for controlled release could be the method of choice: The biomaterials degrade during the drug release, therefore, there is no need for a second surgery to remove the drug eluting agent. Even non-resorbable bone materials are available (e.g., hydroxyapatite (HA)) or resorbable bone graft materials (e.g., beta-tricalcium phosphate (β-TCP)) that will be replaced by newly formed bone. Composite materials with organic additives (e.g., collagen) supports the handling during surgery and enhances the drug loading capacity, as well as the drug releasing time. The purpose of this study was to investigate the loading capacity and the release rate of Vancomycin and Gentamicin on TCP and HA granules in the shape of a degradable scaffold compared to composite materials from TCP mixed with porcine collagen. Its antibacterial efficacy to a more elementary drug with eluting in aqueous solution was examined. The loading capacity of the biomaterials was measured and compared according to the Minimum Inhibition Concentration (MIC) and the Minimum Biofilm Eradication Concentration (MBEC) of a bacterial biofilm after 24 h aging. Antibiotic elution and concentration of gentamycin and vancomycin, as well as inhibition zones, were measured by using the Quantitative Microparticle Systems (QMS) immunoassays. The antibiotic concentration was determined by the automated Beckman Coulter (BC) chemistry device. For examination of the antibacterial activity, inhibition zone diameters were measured. Generally, the antibiotic release is more pronounced during the first couple of days than later. Both TCP granules and HA granules experienced a significantly decline of antibiotics release during the first three days. After the fourth day and beyond, the antibiotic release was below the detection threshold. The antibiotic release of the composite material TCP and porcine collagen declined less drastically and was still in the frame of the specification during the first nine days. There was no significant evidence of interaction effect between antibiotic and material, i.e., the fitted lines for Gentamycin and Vancomycin are almost parallel. During this first in vitro study, β-TCP-Collagen composites shows a significantly higher loading capacity and a steadily release of the antibiotics Gentamycin and Vancomycin, compared to the also used TCP and HA Granules.

## 1. Introduction

The treatment of osteomyelitis is still facing challenging problems in orthopedic surgery. By reaching a chronic state of osteomyelitis, the local effectiveness of systemic anti-infectives is reduced due to a diminished local vascularity [1]. Additionally, a delayed treatment of osteomyelitis leads to osseous resorption [2]. 

Different methods and various grafting materials are used for the purpose of bone reconstruction [3]. Among the bone graft materials, synthetic calcium phosphate bone graft materials, with excellent biocompatibility, are commonly used as alternatives to autogenous bone or xenograft, or allograft materials [4,5]. 

Collagen-ceramic composites as synthetic bone regeneration materials have been developed for non-weight-bearing applications and the addition of collagen confers a number of advantages. These are built upon the material properties of the composite having the hemostatic function that allows early wound stabilization, attracts fibroblasts, and facilitates nutrient transport [6,7]. As the collagen matrix also binds blood clots with regenerating bone cells from the host bone, it has proven to accelerate bone regeneration [7].

The local application of antibiotics, besides surgical debridement, represents a viable alternative treatment to handle osteomyelitis by reaching higher local antibiotic concentrations and minimizing systemic side effects [8]. 

Gentamicin and Vancomycin are common antibiotics in use for the treatment of bacterial infections. Because of the increasing resistance vancomycin is frequently used because of its antimicrobial activity against Gram-positive bacteria together with gentamicin against gram-negative bacteria as main targets [9]. Both substances are also in use in combination with polymeric bone cements (polymethylmethacrylate (PMMA)) for controlled release of the pharmaceutical agents by implanting prosthetic devices in patients. The concentration of both substances was 2.5%. Serum levels of <0.2 to 1.0 mg/L (µg/mL) gentamicin was measured and 13.8–40 mg/mL vancomycin [10].

For the successful treatment of the infection, particularly when glycosides were used [11], is the amount of the active agent released from the donor decisive in order to reach the minimum inhibitory concentration (MIC) [12]. It is considered that a concentration 8–10 times higher than the MIC would be required for an effective antibiotic function [13]. The MIC is estimated at 1 µg/mL for Gentamycin and 2 µg/mL for Vancomycin. After 24 h a biofilm is formed. The Minimum Biofilm Eradication Concentration (MBEC) is up to 51,200 times higher than MICs. The MBEC of Gentamycin is 6400 µg/mL and of Vancomycin 3200 µg/mL [14].

Van de Belt described the release kinetics from polymethylmethacrylate bone cements as a combination of surface properties and porosity, which shows a biphasic process (burst-sustained release) in a study [15]. After a strong initial release of antibiotics follows a phase of significantly reduced elution- and antimicrobial capacity. Under these conditions, an increase of porosity, as it is achieved with the collagen-ceramic-composite, generally leads to a more effective active agent release.

The intention of this in vitro study was a comparing examination of different antibiotics in combination with commercially available bone graft substitutes. The antibiotics, as well as the biomaterials, do have different structures and therefore the combination of both are different in dependence with the material (β-TCP, HA, Collagen), the structure (inner surface, porosity) and pharmaceutical agent. The preparation and the uptake of the antibiotic formulations under clinical conditions were simulated by dripping the solutions onto the biomaterials and measuring the loading capacity. The loading capacity was measured in order to understand which amount of antibiotics can be combined for clinical applications. For drug release experiments, the biomaterials were eluted in aqueous solution, and the concentration of the pharmaceutical active substance was measured during elution time in the surrounding liquid. Two experimental setups were implemented in order to measure the release behavior of different Gentamycin and Vancomycin-doped ceramic which was based on bone substitute (β-TCP granules with low porosity, HA granules with high porosity, and a β-TCP-collagen composite). The conventional test setup is dipping the loaded biomaterials in an aqueous solution and measuring the drug release as a function of time by measuring the concentration in the solution. This is a dilution method to understand the interaction between antibiotic and biomaterial. Another test method is dripping the above described solution onto an agar plate with bacterial layer by measuring the inhibition zone around the laden biomaterial as a function of time. So, the antibacterial activity and function of the antibiotics, first laden on the biomaterial and then released in the aqueous solution, is monitored.

## 2. Materials and Methods

### 2.1. Drug Eluting Matrices

Open cellular hydroxyapatite (HA) granulate with a porosity of 75% (Osbone^®^) and β-tricalcium phosphate granulate with a porosity of 65% (Cerasorb^®^ M) were both used with a granulate size of 1000–2000 µm. β-tricalcium phosphate composite materials consisted of 85 vol % porcine collagen and 15 vol % β-tricalcium phosphate (Cerasorb^®^ Flexible Foam) with a density of (0.4 ± 0.1) g/cm^3^ was used in this work. Depending on its density, the materials had different mechanical characteristics. All materials were provided by Curasan AG, Kleinostheim, Germany.

### 2.2. SEM Examination and Materials Characterization

The scaffolds were examined by Scanning Electron Microscopy (SEM) in order to determine the surface and porosity characteristics of the biomaterials. The samples were placed on a sample holder using conductive carbon tape followed by sputtering with graphite and gold. The materials were examined using an AMRAY Scanning Electron Microscope Type 1810P (SEMTech Solutions Inc., 6 Executive Park Drive, North Billerica, MA, USA). Photos were filed using different magnifications depending on the surface characteristic of the material. The pore sizes were measured using the scale bars. The density and porosity of the materials was determined by measuring the bulk density of the scaffolds.

### 2.3. Antibiotics Measurement Assay

The QMS Gentamicin and Vancomycin assays are homogeneous particle-enhanced turbidimetric immunoassays. The assays are based on competition between drug in the sample and drug coated onto a microparticle for antibody binding sites of the antibody reagents. The antibiotic-coated microparticle reagent is rapidly agglutinated in the presence of the anti-antibody reagent and in the absence of any competing drug in the sample. The rate of absorbance change is measured photometrically. When a sample containing the antibiotic is added, the agglutination reaction is partially inhibited, slowing down the rate of absorbance change. A concentration-dependent classic agglutination inhibition curve can be obtained with maximum rate of agglutination at the lowest antibiotic concentration and the lowest agglutination rate at the highest antibiotic concentration. The antibiotic concentration was determined by the automated Beckman Coulter AU480-clinical chemistry device (Beckmann Coulter, Brea, CA, USA).

### 2.4. Antibiotics Uptake Preparation

For each experiment there was 100 mg biomaterial used. Cerasorb^®^ Foam pellets were cut from the scaffolds. The granules of biomaterials were used in the original structure. Therefore, five samples of every sort of the respective bone graft substitutes Cerasorb^®^ M granules (overall porosity 65%), Cerasorb^®^ Foam (overall porosity 80%), and Osbone^®^ granulate (overall porosity 75%) were soaked with 4 mL of the antibiotic solutions (Gentamicin-Ratiopharm 80 SF (4% solution), ratiopharm GmbH, Ulm, Germany, 40 mg/mL); and Vancomycin 50 mg/mL (5% solution), Hikma Pharma GmbH, Gräfelfing, Germany) for 1 min at room temperature in a petri dish under simulated clinical conditions.

### 2.5. Measurement of Total Antibiotics Uptake

The total antibiotic uptake of the pellets was carried out by pestling the pellets and loading with antibiotics. Subsequently, 5 mL PBS was also added and followed by incubation for 24 h at 37 °C. The supernatant was measured with the Beckman Coulter AU480 device as described in Section 2.3. In order to calculate the real antibiotic uptake of the scaffolds in wt % (m_Antibiotic in wt %_), the following Equation (1) was used:

(1) $$m_{Antibiotic\;in\;wt\;\%}=\frac{5\;\times \;c_{Beckman\;Coulter}\;\times \;m_{Biomaterial}}{100,000},$$

with: *c_Beckman Coulter_*—Measurement of AU480 in mg/L in 5 mL PBS substrate, and *m_Biomaterial_*—Initial weight of the biomaterial sample.

From these data, the minimum biomaterial amount with uptaken antibiotics to reach the MBEC, according to Marques et al., in mg was calculated [14].

### 2.6. Measurement of the Release Kinetics

The release kinetics of the antibiotics Gentamicin and Vancomycin with regard to the bone graft substitutes was determined. The released antibiotic concentrations were measured using the Quantitative Microparticle Systems (QMS) Gentamicin and Vancomycin immunoassays (Thermo Scientific^TM^, Rockford, IL, USA). The detection limit for the QMS was 2.5 µg/mL for Vancomycin and 0.5 µg/mL for Gentamycin. Additionally, disk diffusion was used to measure the effect of the antibiotic release on microorganisms. 

After the inoculation step, the supernatant was discarded and the pellets were incubated in 10 mL phosphate-buffered saline (PBS), pH 7.4, at 37 °C for 24 h. The release kinetics of each of the two antibiotics was measured in multiple test runs (n = 5) on 10 consecutive days. Each day, the supernatant was taken from the solution. One hundred microliters was used for disk diffusion, and the rest of the solution was preserved at −80 °C for subsequent determination of the antibiotic concentration. The pellets were washed with PBS and then, again, 10 mL PBS were added, followed by subsequent incubation.

Outgoing from the release percentage after 24 h the minimum amount of Antibiotic-loaded biomaterial was calculated to reach MBEC after 24 h.

For disk diffusion, a bacterial suspension of the test organisms Bacillus subtilis ATCC 6633 and Staphylococcus aureus ATCC 25923 was adjusted using a McFarland standard of 0.5 in 0.9% sterile saline and streaked over the whole surface of a Columbia blood agar plate with a sterile swab. One hundred microliters of the supernatant of the incubated bone graft substitutes was transferred in the middle of the plate. After overnight incubation at 35 ± 2 °C, the zone diameters were measured.

### 2.7. Statistical Methods

In order to describe the antibiotics release as a function of time, two evaluation methods were used. Outgoing from the Antibiotic concentrations in the supernatant measured by the Beckman Coulter Device (Beckmann Coulter, Brea, CA, USA) in mg/L. 100%-release of the drug was supposed when no change of the amount was detected. The release was then plotted in cumulative percent release. In a second method, piecewise linear functions to the antibiotics release were fitted, i.e., $\log_{10}\left(antibotics\;release\right)=\;\{\begin{array}{c} \;\beta_{0}+\;\beta_{1}\times \mathrm{day}\;\mathrm{for}\;\mathrm{day}<d_{0}\\ \beta_{0}^{\prime }+\;\beta_{1}^{\prime }\times \mathrm{day}\;\mathrm{for}\;\mathrm{day}\geq \;d_{0} \end{array}$, using the NLMIXED procedure of the diagnostic program of SAS^®^ Analytic Software (Version 9.4, SAS Institute GmbH, Heidelberg, Germany) and Solutions to fit nonlinear fixed models. Multiple measurements on each specimen were taken into account by including a random specimen effect in the model.

In order to describe the dependence of the inhibition zones for staphylococcus aureus and bacillus subtilis on antibiotics release, a linear mixed model (SAS PROC MIXED) was used, taking multiple measurements on each specimen into account by including a random specimen effect in the model. The dependent variable was the area of the inhibition zone, which was calculated from the diameter, assuming a circular shape. Independent variables were antibiotics’ release, material, and type of antibiotic. Here, observations with antibiotics release below the detection threshold were excluded from the model, as these observations neither had a detectable inhibition zone.

Antibiotic uptake was compared, using the Kruskal–Wallis test and Wilcoxon–Mann–Whitney test, and exact p-values were determined. All analyses were performed using SAS 9.4.

## 3. Results

### 3.1. SEM Examination

**Figure 1:**
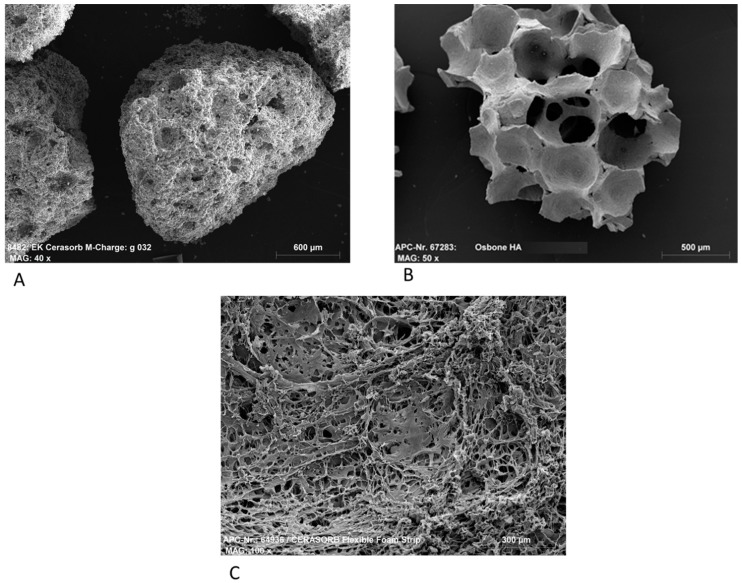
SEM photos of (**A**): Cerasorb^®^ M, Magnification 40×, (**B**): Osbone^®^, Magnification 50×, and (**C**): Cerasorb^®^ Flexible Foam Strip (average density 0.4 g/cm^3^), Magnification 100×.

Figure 1 shows the structure of the bone regeneration materials in the Scanning Electron Microscope. Cerasorb^®^ M showed a rough surface with statistical distributed pores from 0.2–500 µm. The inner surface is enhanced by this interconnecting micro-, meso-, and macroporosity.

Osbone^®^ shows a bone-like structure. Pores are replaced by open cellular structure formed by bars of 10–50 µm thickness. These bars are formed by the primary HA particles forming a bone-like nearly 80% porous structure with a medium inner surface.

Cerasorb^®^ Foam shows a fibrous structure of collagen fibers. The ceramic is completely embedded in this structure and is not visible by this examination method. The material is open-structured, highly porous, and has a maximized inner surface. Due to the fibrous structure of the organic phase, the material has a high internal liquid uptake characteristic.

### 3.2. Antibiotics Uptake

For each antibiotic, there is a clear distinction in the uptake, with Cerasorb^®^ Granules having the lowest uptake, Osbone^®^ Granules an intermediate intake, and Cerasorb^®^ Foam having the highest intake (for all pairwise comparisons *p* = 0.008). One measuring value with Cerasorb^®^ Foam was an outlier value and, therefore, discarded from the analysis. When comparing antibiotics, no difference in uptake could be demonstrated for Cerasorb^®^ Foam and Osbone^®^ Granules. However, for Cerasorb^®^ Granulate the Vancomycin uptake was clearly higher than the Gentamycin uptake (Table 1).

The complete antibiotics release was measured using 100 mg biomaterial. The amount of active pharmaceutical agent was measured in 5 mL solution as µg/mL. The calculated minimum amounts of biomaterial laden with antibiotics are listed in Table 2. Additionally, the amount of antibiotic release after 24 h is listed, showing that the MIC was reached in all cases. To reach the MBEC, 133–1200 mg biomaterial was necessary (Table 3). To fill an average bone defect, these minimum amounts would be clearly exceeded.

### 3.3. Antibiotic Release in Aqueous Solution

**Figure 2:**
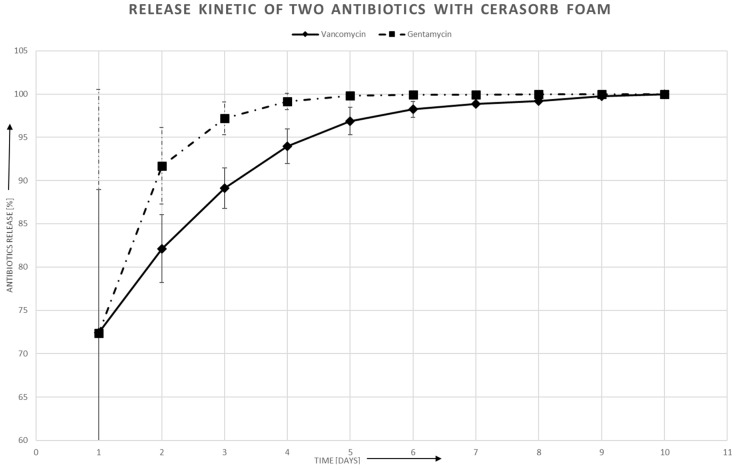
Release kinetics of Vancomycin and Gentamycin from Cerasorb^®^ Foam.

**Figure 3:**
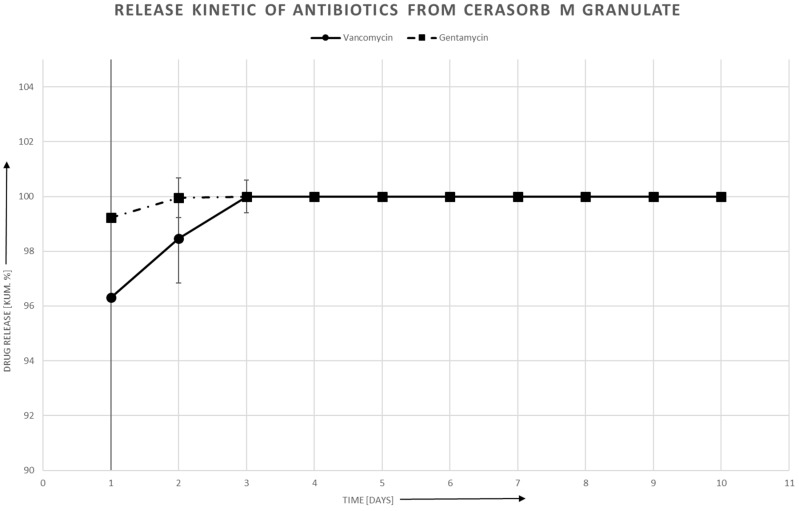
Release kinetics of Vancomycin and Gentamycin from Cerasorb^®^ M.

**Figure 4:**
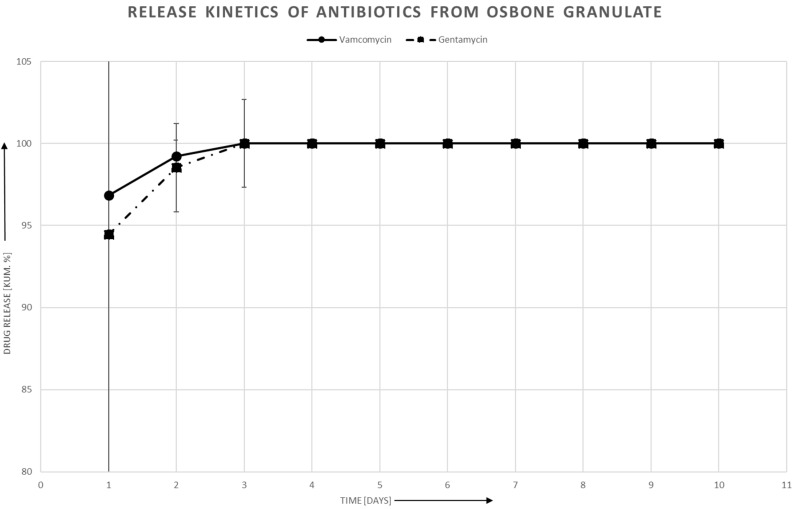
Release kinetics of Vancomycin and Gentamycin from Osbone^®^.

The antibiotics release was faster with the granulates than with the Cerasorb^®^ Foam. Figure 2 shows the release of Gentamycin and Vancomycin with the Collagen composite material, showing that the antibiotics release is faster with Gentamycin. In all cases, an initial burst can be seen, stronger with the ceramic granulates than with the collagen composite material. The deviation in the first two days is higher than in the later stages where results and deviation become more comparable, whereas at the end at the release of nearly 100%, no deviation can be detected. The release ends after six days with Gentamycin, whereas Cerasorb^®^ Foam loaded with Vancomycin has an end release kinetic of 10 days. Both Granulates, Cerasorb^®^ M and Osbone^®^, showed a faster release kinetic and 100% of the drug was released after two days (Figure 3 and Figure 4). The fitted piecewise linear functions revealed a change point at 2.76 days (Table 4 and Table 5). So, the antibiotics release for each combination of antibiotic and material can be described by one linear regression equation for the first three days and by another linear regression equation for the following days. 

**Figure 5:**
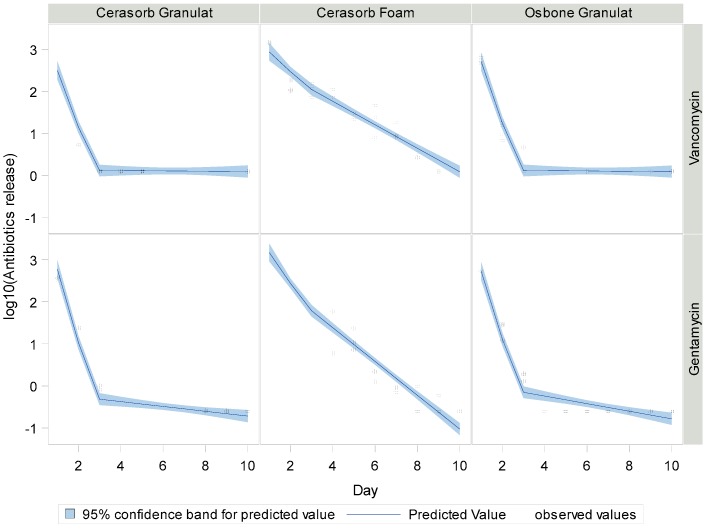
Logarithmic draw and linear fit of drug release kinetics.

Generally, the slope is steeper in the first days than later on for all biomaterials. Table 4 and Table 5 show that the difference with Cerasorb^®^ Foam is only 0.2 to 0.3 for both antibiotics. There is a significant difference in intercept and slope between Gentamycin and Vancomycin with the granular biomaterials, with Gentamycin having on average 0.67 higher intercepts (*p* = 0.009), steeper slopes in the first part (difference = −0.40, *p* = 0.0008), and more gentle slopes in the second part (difference 0.34, *p* = 0.008). There was no significant interaction effect of antibiotic and material, i.e., the fitted lines for Gentamycin and Vancomycin are almost parallel (Figure 5).

In the first day, the MIC is immediately reached for all material and antibiotic combinations. An acute inflammation reaction caused by bacterial infection would be threatened effectively under clinical conditions. For both, Cerasorb^®^ Granulate and Osbone^®^ Granulate, there is a steep decline of antibiotics release in the first three days. From day four onwards, the antibiotics release is below the detection threshold. For Cerasorb^®^ Foam, the decline is less steep and, up to day nine in at least some of the specimens, antibiotics are still released.

Outgoing from the percentage of antibiotics release after one day, the minimum amount of biomaterial with pharmaceutical agent was calculated (Table 6). It can clearly be seen that the loading capacity of all bone graft substitutes is applicable and only a minimum amount of 400 mg up to 2 g is enough to reach a minimum dose for biofilm treatment [14]. The maximum load listed in Table 2 and the release percentage and amount in Table 3 shows the comparability of the measurements.

### 3.4. Inhibition Zones

#### 3.4.1. Staphylococcus Aureus

For staphylococcus aureus, there is a roughly linear relationship between area of the inhibition zones and log (antibiotics release). However, there may exist a threshold, especially in Cerasorb^®^ Foam, for the antibiotics release, such that no inhibition zone can be established if the antibiotics release is below this threshold.

**Figure 6:**
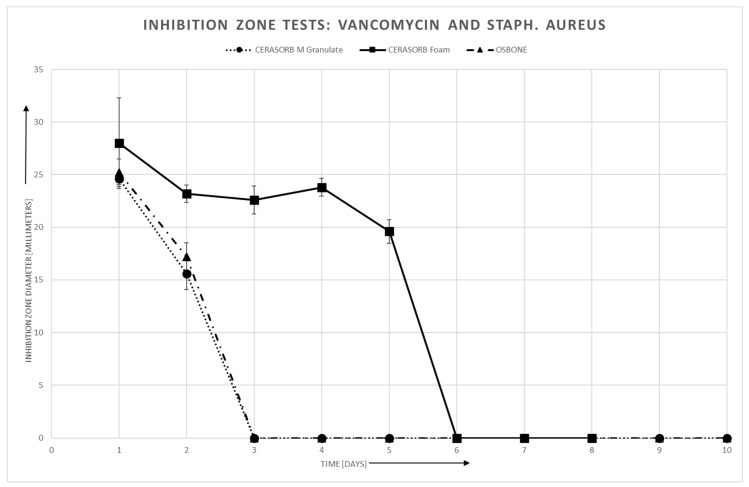
Vancomycin and Staphylococcus aureus: Inhibition zone area vs. time.

In Gentamycin, the linear relationship is quite similar for all three materials, whereas in Vancomycin, the slope is steeper for Cerasorb^®^ Form compared to the granules (Figure 5 and Figure 6). 

#### 3.4.2. Bacillus Subtilis

For bacillus subtilis, there is also a roughly linear relationship between area of the inhibition zones and log (antibiotics release). Again, there may exist a threshold, especially in Cerasorb^®^ Foam, for the antibiotics release, such that no inhibition zone can be established if the antibiotics release is below this threshold.

**Figure 7:**
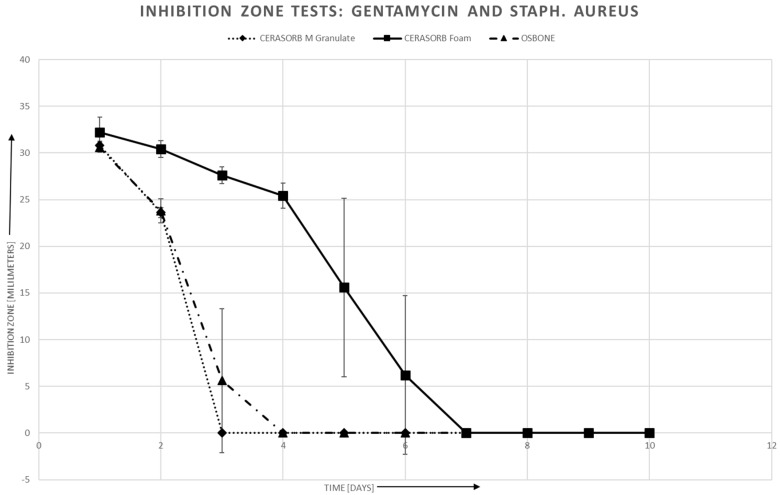
Gentamycin and Staphylococcus aureus: Inhibition zone area vs. time.

**Figure 8:**
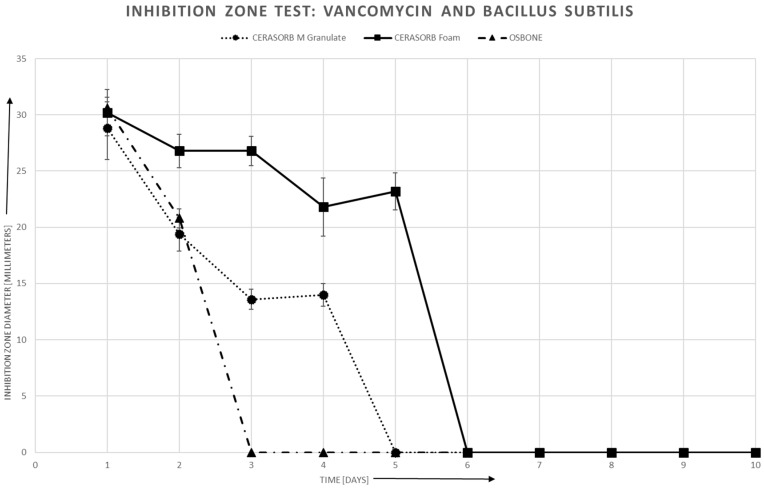
Vancomycin and Bacillus subtilis: Inhibition zone area vs. time.

**Figure 9:**
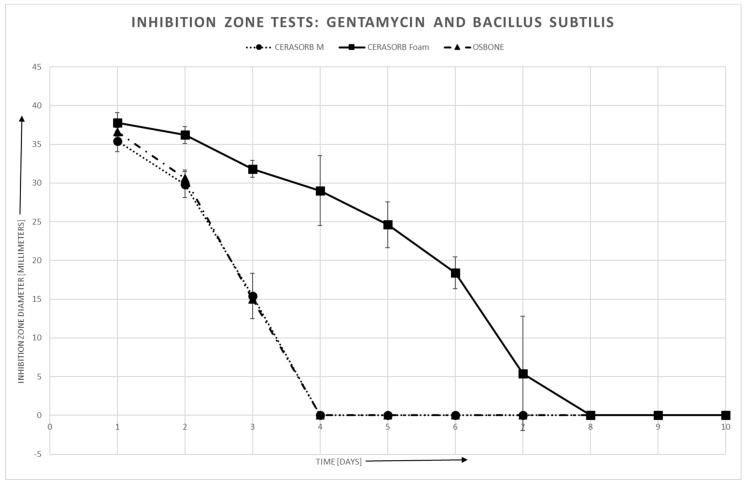
Gentamycin and Bacillus subtilis: Inhibition zone area vs. time.

Like for staphylococcus aureus, the linear relationship is quite similar for all three materials in Gentamycin, whereas in Vancomycin, the slope is steeper for Cerasorb^®^ Foam than for the granulates (Figure 7, Figure 8 and Figure 9).

## 4. Discussion

The idea to dope resorbable bone graft substitutes with antibiotics for treating Osteomyelitis was realized by several researchers. McKee reported about similar eradication rates by Tobramycin, released out of calcium sulfate (CS) pellets, in comparison to polymethylmethacrylate (PMMA) beads [16,17]. Pure ceramic materials, like tricalcium phosphate (TCP) or hydroxyapatite (HA), which are easy to load with antibiotic liquids but also release them quick and over a short period of time with a resulting minimal inhibition concentration (MIC) only for a few days [18,19], were also discussed. 

In a previous work, different biomaterials, amongst others also Cerasorb^®^ M Granules and the Collagen composite Cerasorb^®^ Foam, were loaded with different concentrations of Vancomycin solution from the same origin like in this work. As distinguished from this work the biomaterials were loaded with a dripping technique under normal conditions as well as in vacuum to obtain a more and a complete soaking of all pores and interstices of the biomaterials. The incubation was then carried out in a drying oven at 50 °C for 96 h. Due to this dripping and drying technique a higher load of pharmaceutically active substance could be reached. The release kinetic did not show the strong initial bursts like in this actual work and had a much longer release kinetic: even after 14 days incubation period nearly 50% of the antibiotic remained in the biomaterial caused by a stronger binding of the substance on the inner surface of the biomaterials. With this way the antibiotic concentrations around the materials are at a nearly constant level over long periods and could be helpful for clinical applications. The remaining active substance will be released during the biodegradation/resorption of the biomaterials. Additional experiments of controlled drug release with Vancomycin from these biomaterials under lower pH conditions, simulating the treatment of infected areas showed an irregular release kinetic [20]. In this actual work commercially available biomaterials were combined with antibiotic preparations under clinical conditions without vacuum and/or drying steps at higher temperatures. The initial burst of concentration was higher, and the release of the pharmaceutically active substance was nearly completed in a much shorter period. The loading capacity can be confirmed for both Gentamycin and Vancomycin. Compared to granules of different porosities, a significantly higher bonding of the antibiotics to the substitutes matrix, a higher and longer release rate, and a more sustainable efficacy in the zone of inhibition testing could be observed. 

The good adsorption to the composite matrix can be explained by multiple effects: On the one hand, the surface of collagen is much bigger than it is in Calcium phosphates (0.1–0.2 m^2^/g vs. 80 m^2^/g). This opens a huge potential for physisorption of the antibiotics. The big amount of functional side chains in collagen with polar COOH- and NH_2_- groups should also provide significant ionic and electronic interdependencies. The question of why Vancomycin, compared to Gentamycin, demonstrates a stronger bonding to the provided matrix can obviously be answered by the higher molecular weight of Vancomycin (1450 vs. 450 Dalton). 

In 1997, Stemberger et al. reported about high local levels and low serum antibiotics after implantation of collagen–antibiotic combinations [21]. Comparing the gentamicin pharmacokinetics in the zone of subcutaneous implantation around polymethylmethacrylate beads, Firsov et al. observed a three-phase pattern for collagen sponges [22]: [i] An initial high antibiotic peak, which is in accordance with the microbiological demands of high doses for a short time; [ii] after a few h, stabilization of the gentamicin concentration to a practically constant level, above the minimal inhibitory concentration (MIC) of many pathogens for several days; and [iii] finally, the antibiotic level in the tissue diminishes [23]. Comparing the available results due to the extended active agent release out of the granule-collagen-composite, confirmed by the study of Firsov with the accelerated release of the granules, we assume that the collagen part in the composite formulation has a key role for the extended release. It supports the theory that the local drug levels are very much dependent on the application site [24]. The concept of a diffusion model circulates widely, in which the porosity of the donor site is responsible for the magnitude of release. According to this thesis, the surface-to-volume ratio is considered as decisive for the extent of the release of the agent from the donor matrix, with the size and dimensions of the pores acting as an indicator of the emission capacity [25]. It is agreed that increasing the surface area of the donor and the improved transport of fluids that results, along with the permeability, has an influence on the release kinetics of antibiotics [26]. Following the interpretation of the authors, the open cell structure of the collagen is responsible for the represented pharmacokinetics. The degradation characteristics for TCP biomaterials, as well as collagen products, was extensively studied [3,6]. During the observation period, the pharmaceutical agent was completely eluted and biodegradation played only a minor role for this release kinetic.

The single use of collagen matrices seems to show similar characteristics in release kinetics as it is observed for granules and beads [27]. Obviously, just the combination with TCP provides the long-term release. Comparable effects are also known from coating the collagen scaffold with polyester [23].

The loading capacity of all biomaterials used in this study is suitably high and, therefore, a minimum amount of each biomaterial loaded with Gentamycin or Vancomycin is necessary to reach the MIC. Even in 100 mg Biomaterial, the 500- to 2.500-fold capacity is provided (Table 2). For the treatment of a biofilm that has formed after 24 h 0.1–1.2 g Biomaterial, laden with antibiotics, is necessary to reach the MBEC, depending on the type of antibiotic and matrix (Table 3). The results, detected in this work, can be used for calculating an effective treatment. The measurements of uptake capacity and drug eluting amounts are comparable and show the accuracy of the results.

Aqueous environments used in this drug release model lead to a faster release than it is to be expected in a biological environment like in a bone defect. It can be, therefore, concluded that the MIC will be maintained after surgery for more than the time measured in this study. After release, the antibiotic is still in the surrounding tissue and will not decompose at once. An in vivo environment is humid, and contains cells and enzymes that could also affect the implanted material. Cells might also take up the antibiotic and deplete it. For developing these effects, an in vivo model is necessary that allows monitoring of the antibiotic concentration in the organism and preferably also at the site of operation. Several in vivo models, such as sclerotherapy, open fractures, or segmental defects, are proposed [9]. In a next step, the combination of the biomaterials used in this study should be tested in vivo according the descriptions in this review to reach comparable results. Finding a method of in vitro drug release under similar conditions, like in animal models, could help to understand the measurements in serum and other fluids [10].

## 5. Conclusions

All biomaterials used in this study showed a suitable loading capacity of the two antibiotics, a minor quantity of antibiotic is needed to reach the specification of MIC (Minimum Inhibition Concentration), while a larger quantity is needed to reach the MBEC (Minimum Biofilm Eradication Concentration). In all cases, the drug-released kinetic started with an initial burst reaching the MIC in the surrounding liquid in the first 24 h. A slower release of the pharmaceutical agents took place in a second step. The drug release of the granulated material was faster compared to the collagen composite material. In all cases, the pharmaceutical agent was practically released to 100%. 

The antibacterial activity of the antibiotics were measured after the loading and release from the biomaterials, which was still showing an inhibitory effect of Staph. Aureus and Bacillus subtilis. β-TCP-Collagen Composites showed in this first in vitro study a significantly stronger and longer release of Gentamycin and Vancomycin compared to β-TCP- and hydroxyapatite granules. For clinical use, a more sustainable therapy against sensitive exciters is to be expected. The osteoconductivity of the composite may offer an additional positive effect in the treatment of bone infections.

## Figures and Tables

**Table 1:** Antibiotics uptake by material and type of antibiotic.

-	Antibiotics Uptake in wt %			
-	Gentamycin		Vancomycin	
-	Mean	Standard Deviation	Mean	Standard Deviation
Osbone^®^ Granulate	6.50	1.18	7.48	0.86
Cerasorb^®^ M Granulate	2.65	0.56	5.15	1.00
Cerasorb^®^ Foam	12.69	2.95	12.08	4.05

**Table 2:** Antibiotic release after 24 h showing that the Minimum Inhibitory Concentration (MIC) is reached in all cases in early stages, mean antibiotics uptake, and calculated minimum amount of biomaterial to reach the minimum antibiotic concentration to erase a biofilm (MBEC) by material and type of antibiotic.

-	Gentamycin			Vancomycin		
-	Mean Release after 24 h (µg/mL)	Mean Uptake (µg/mL)	Biomaterial Amount to Reach MBEC (mg)	Mean Release after 24 h (µg/mL)	Mean Uptake (µg/mL)	Biomaterial Amount to Reach MBEC (mg)
Osbone^®^ Granulate	512	1295	494	571.6	1496	214
Cerasorb^®^ M Granulate	460.8	530	1208	446	1032	310
Cerasorb^®^ Foam	1424.2	2540	252	1184	2408	133

**Table 3:** Minimum amount of antibiotic-loaded biomaterial to reach MBEC after 24 hours for bacterial biofilm treatment calculated from release kinetic.

-	Gentamicin		Vancomycin	
-	% after 24 h	Minimum Amount to Reach MBEC (mg)	% after 24 h	Minimum Amount to Reach MBEC (mg)
Osbone^®^ Granules	94.5	467	96.8	207
Cerasorb^®^ M Granules	99.2	1198	96.3	299
Cerasorb^®^ Foam	72.3	182	72	96

**Table 4:** Regression equations describing the Gentamycin release over time.

Material	Condition	Equation
Cerasorb^®^ Foam	if day ≤ 2.761	log_10(*Antibiotics release*)_ = 3.905–0.733 × day
Cerasorb^®^ Foam	if day > 2.76	log_10(*Antibiotics release*)_ = 2.991–0.402 × day
Cerasorb^®^ Granules	if day ≤ 2.76	log_10(*Antibiotics release*)_ = 4.524–1.748 × day
Cerasorb^®^ Granules	if day > 2.76	log_10(*Antibiotics release*)_ = −0.144–0.057 × day
Osbone^®^ Granules	if day ≤ 2.76	log_10(*Antibiotics release*)_ = 4.349–1.622 × day
Osbone^®^ Granules	if day > 2.76	log_10(*Antibiotics release*)_ = −0.085–0.016 × day

**Table 5:** Regression equations describing the Vancomycin release over time.

Material	Condition	Equation
Cerasorb^®^ Foam	if day ≤ 2.76	log_10(*Antibiotics release*)_ = 3.407–0.470 × day
Cerasorb^®^ Foam	if day > 2.76	log_10(*Antibiotics release*)_ = 2.882–0.280 × day
Cerasorb^®^ Granules	if day ≤ 2.76	log_10(*Antibiotics release*)_ = 3.850–1.353 × day
Cerasorb^®^ Granules	if day > 2.76	log_10(*Antibiotics release*)_ = 0.124–0.003 × day
Osbone^®^ Granules	if day ≤ 2.76	log_10(*Antibiotics release*)_ = 4.169–1.468 × day
Osbone^®^ Granules	if day > 2.76	log_10(*Antibiotics release*)_ = 0.125–0.004 × day

**Table 6:** Minimum amount of antibiotic-loaded biomaterial to reach MBEC after 24 h for bacterial biofilm treatment calculated from release kinetic.

-	Gentamicin		Vancomycin	
-	% after 24 h	Minimum Amount to Reach MBEC (mg)	% after 24 h	Minimum Amount to Reach MBEC (mg)
Osbone^®^ Granules	94.5	467	96.8	207
Cerasorb^®^ M Granules	99.2	1198	96.3	299
Cerasorb^®^ Foam	72.3	182	72	96
